# Characterization of two diesel fuel degrading microbial consortia enriched from a non acclimated, complex source of microorganisms

**DOI:** 10.1186/1475-2859-9-10

**Published:** 2010-02-16

**Authors:** Giulio Zanaroli, Sara Di Toro, Daniela Todaro, Giovanna C Varese, Antonio Bertolotto, Fabio Fava

**Affiliations:** 1 DICASM, Faculty of Engineering, University of Bologna, via Terracini 28, 40131 Bologna, Italy; 2 MARCOPOLO ENGINEERING Spa, via XI Settembre 37, 12011 Borgo San Dalmazzo (CN), Italy; 3 Department of Plant Biology, University of Torino, Viale Mattioli 25, 10125 Torino, Italy

## Abstract

**Background:**

The bioremediation of soils impacted by diesel fuels is very often limited by the lack of indigenous microflora with the required broad substrate specificity. In such cases, the soil inoculation with cultures with the desired catabolic capabilities (bioaugmentation) is an essential option. The use of consortia of microorganisms obtained from rich sources of microbes (e.g., sludges, composts, manure) via enrichment (i.e., serial growth transfers) on the polluting hydrocarbons would provide bioremediation enhancements more robust and reproducible than those achieved with specialized pure cultures or tailored combinations (co-cultures) of them, together with none or minor risks of soil loading with unrelated or pathogenic allocthonous microorganisms.

**Results:**

In this work, two microbial consortia, i.e., ENZ-G1 and ENZ-G2, were enriched from ENZYVEBA (a complex commercial source of microorganisms) on Diesel (G1) and HiQ Diesel (G2), respectively, and characterized in terms of microbial composition and hydrocarbon biodegradation capability and specificity.

ENZ-G1 and ENZ-G2 exhibited a comparable and remarkable biodegradation capability and specificity towards n-C10 to n-C24 linear paraffins by removing about 90% of 1 g l^-1 ^of diesel fuel applied after 10 days of aerobic shaken flask batch culture incubation at 30°C. Cultivation dependent and independent approaches evidenced that both consortia consist of bacteria belonging to the genera *Chryseobacterium*, *Acinetobacter*, *Psudomonas*, *Stenotrophomonas*, *Alcaligenes *and *Gordonia *along with the fungus *Trametes gibbosa*. However, only the fungus was found to grow and remarkably biodegrade G1 and G2 hydrocarbons under the same conditions. The biodegradation activity and specificity and the microbial composition of ENZ-G1 and ENZ-G2 did not significantly change after cryopreservation and storage at -20°C for several months.

**Conclusions:**

ENZ-G1 and ENZ-G2 are very similar highly enriched consortia of bacteria and a fungus capable of extensively degrading a broad range of the hydrocarbons mainly composing diesel fuels. Given their remarkable biodegradation potential, stability and resistance to cryopreservation, both consortia appear very interesting candidates for bioaugmentation operations on Diesel fuel impacted soils and sites.

## Background

Diesel fuels are complex mixtures of saturated hydrocarbons (primarily paraffins including *n*, *iso*, and cycloparaffins), and aromatic hydrocarbons (including naphthalenes and alkylbenzenes) obtained from the middle-distillate, gas-oil fraction during petroleum separation. Due to their massive production and use as fuels for transportation, they are among the most common sources of organic pollutants for the surface soil. They also impact the subsurface soil through leaking from underground storage tanks and pipelines. Due to their mobility in soil, such released diesel fuel hydrocarbons can reach water intakes and/or groundwater reservoirs, thus generating relevant risks for humans and other living organisms [1].

The majority of petroleum-derived hydrocarbons can be biodegraded by several microbial strains each however capable of breaking down a specific group of molecules; the biodegradation potential generally decreases by moving from n-alkanes to branched alkanes, low-molecular weight n-alkyl aromatics, monoaromatics, cyclic alkanes and polynuclear aromatics [2-4]. However, the mineralization of complex hydrocarbon mixtures such as those composing diesel fuels, usually requires the co-existence and effective cooperation of several specialized microorganisms with complementary substrate specificity [5-7]. Microbial consortia with such physiological and metabolic features might not exist in a soil, in particular if only recently impacted by a diesel fuel release, and this is often the main cause of the poor bio-treatability of diesel fuel contaminated soils and sites. In such cases, the inoculation of the impacted soil with high concentrations of characterized cultures of highly specialized microbes (bioaugmentation) is one of the most promising options for getting its sustainable remediation.

The use of pure cultures and co-cultures of hydrocarbon-degrading microorganisms has been tested with interesting results on hydrocarbons contaminated soils; however, the behaviour and metabolic activity of the applied cultures are very often not those expected and they are often not reproducible [8-11]. Recently, the use of complex microbial sources, such as those occurring in sludge, manure or compost, has been proposed. They generally carry a high diversity of bacteria and fungi with a high genetic and metabolic diversity, along with a variety of essential nutrients that might remarkably contribute to sustain the survival and colonization of allocthonous microbes in the inoculated biotope [12-19]. However, some of such complex microbial sources contain remarkable loads of non-necessary biotope extraneous microbes, along with pathogenic bacteria, such as *Salmonella *spp., *Listeria monocytogenes*, *Campylobacter coli *and *C. jejuni *[20,21], and therefore their application in bioaugmentation strategies might provide environmental risks that should be carefully evaluated. On the contrary, the use of the specialized portion of microflora of such sources would provide higher and more reproducible pollutant mineralization rates and extents with respect to those achievable with pure or tailored mixed cultures of specialized microorganisms [7,9,22]. This is mostly due to the ability of the members of enriched consortia to retain their native ability to cooperate with each other, also with strains apparently without biodegradation capabilities, that might contribute to the growth and activity of those in charge for the final pollutant mineralization [6]. However, the stability of such complex microbial populations in terms of microbial composition and growth and biodegradation behaviour in the contaminated matrices needs to be determined and assessed, also after prolonged maintenance under both growing and storage conditions [7,23,24].

ENZYVEBA, a partially characterized complex consortium of not-adapted microorganisms developed through prolonged stabilization of cow manure [25-28], was recently applied in bioaugmentation studies [15,16] and found capable of intensifying the aerobic remediation of a soil freshly contaminated by two different diesel fuels, i.e. regular diesel fuel (G1) and HiQ diesel fuel (G2), by acting as a source of both exogenous specialized microorganisms and nutrients [16]. Thus, it was of interest to obtain and characterize its native microbial population capable of diesel fuel hydrocarbon degradation. Two highly enriched microbial populations were obtained from ENZYVEBA through 5 sequential transfers in mineral minimal medium with G1 or G2 as growth substrates. They were then characterized in terms of biodegradation extents, rates and specificity, microbial composition and stability upon cryopreservation, in order to determine their actual potential to be applied in bioaugmentation procedures for diesel fuel contaminated sites. This study is one of the few in which two highly performing diesel fuel mineralizing consortia are obtained from composted related matrices and are described; further it is the first one in which the presence and the key role of a fungus in consortia obtained through conventional enrichment procedures for bacteria is documented and characterized.

## Results

### Diesel fuel-degrading consortia obtained from ENZYVEBA

**Figure 1:**
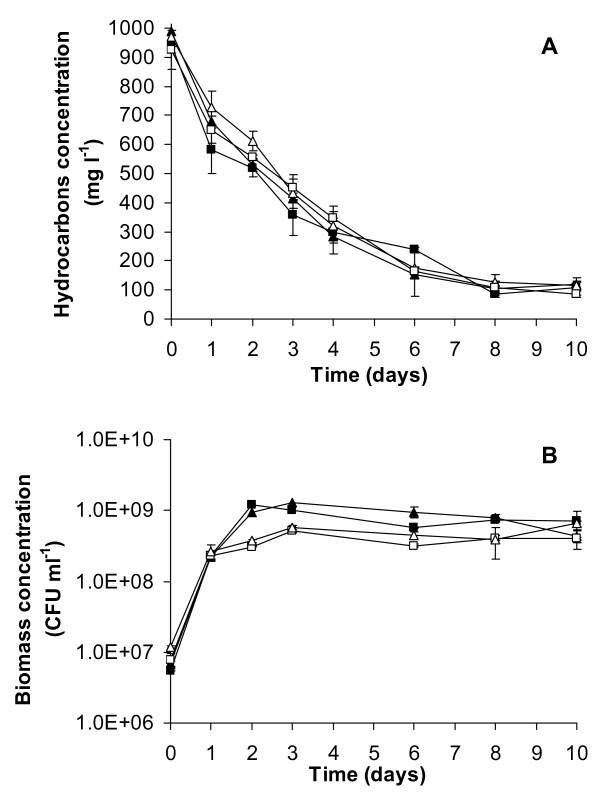
**Biodegradation of hydrocarbons and biomass growth in ENZ-G1 and ENZ-G2 cultures**. Biodegradation of hydrocarbons (A) and concentration of total biomass (B) in ENZ-G1 (squares) and ENZ-G2 (triangles) cultures grown on MMM medium amended with G1 and G2 fuels as the sole carbon and energy source. Solid symbols refer to ENZ-G1 and ENZ-G2 cultures obtained after five enrichment steps; hollow symbols refer to ENZ-G1 and ENZ-G2 cultures after cryopreservation and storage at -20°C for six months followed by three additional sub-culturing cycles.

**Figure 2:**
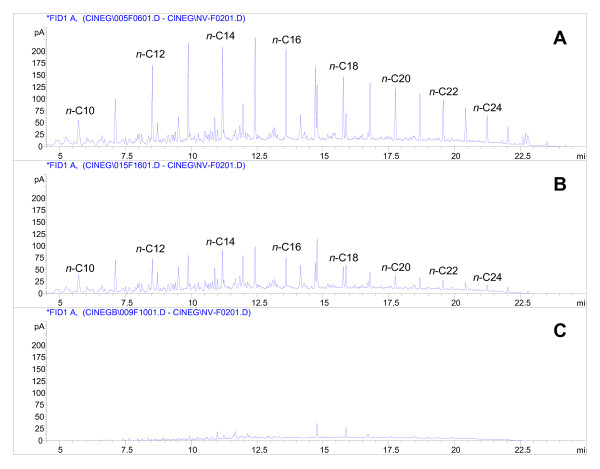
**GC-FID profiles of hydrocarbons obtained from ENZ-G2 culture after 0 (A), 2 (B) and 8 (C) days of incubation**.

The two microbial consortia ENZ-G1 and ENZ-G2 were obtained from ENZYVEBA through 5 consecutive culture transfers (2% v/v) on minimum mineral medium (MMM) for bacteria amended with Diesel (G1) and HiQ Diesel (G2) respectively applied at 1 g l^-1 ^as the main carbon and energy source. Once inoculated in duplicated sterile cultures of MMM with their own diesel fuel at 1 g l^-1 ^in the presence of 20 mg l^-1 ^of yeast extract and incubated at 30°C under shaken flask conditions, both ENZ-G1 and ENZ-G2 degraded rapidly and extensively G1 and G2 hydrocarbons, respectively, with rates which were in average, during the first four days of incubation, 160.9 ± 7.9 and 175.7 ± 3.1 mg of overall hydrocarbons depleted l^-1 ^day^-1^, respectively. The hydrocarbon degradation then proceeded slowly up to the tenth day of incubation, when the initial amount of overall applied hydrocarbons resulted to be removed by 90.8 ± 1.2% and 89.4 ± 0.1% in ENZ-G1 and ENZ-G2, respectively (Figure 1A). The activity of both consortia was mainly directed towards linear C10-C24 paraffins; among these, medium-high MW n-alkanes (C18-C24) were in general degraded more extensively than medium-low MW n-alkanes during the first days of incubation (Figure 2, Table 1). In particular, depletions around 70% and 60% were detected for C18-C24 and C12-C16 n-paraffins, respectively, after 1 day of incubation in ENZ-G1 culture and after 2 days of incubation in ENZ-G2 culture, whereas degradation of 21%-22% was observed for n-decane after two days of incubation (Table 1). However, comparable final depletions of 94%-100% were achieved for all n-paraffines in both cultures at the end of incubation (10 days) (Figure 2, Table 1). No GC-FID detectable intermediates were observed to accumulate in the ENZ-G1 and ENZ-G2 cultures throughout the 10 days of incubation. The bacterial biomass able to grow on TSA medium grew rapidly during the first 3 days of incubation by increasing from 5.4 × 10^6 ^± 7.5 × 10^4 ^and 6.9 × 10^6 ^± 6.0 × 10^5 ^CFU ml^-1 ^observed at the beginning of incubation in ENZ-G1 and ENZ-G2 cultures, respectively, to values of 1.0 × 10^9 ^± 1.5 × 10^7 ^and 1.3 × 10^9 ^± 1.8 × 10^8 ^CFU ml^-1^, respectively (Figure 1B).

**Table 1:** Degradation percentages of linear alkanes by ENZ-G1 and ENZ-G2 cultures

	Degradation percentage - ENZ-G1			Degradation percentage - ENZ-G2		
	
n-alkane	1 day	2 days	10 days	1 day	2 days	10 days
**n-C_10_**	31,2 ± 6,7	22,6 ± 1,6	96,1 ± 2,9	14,1 ± 4,0	21,0 ± 4,3	100,0 ± 0,0
**n-C_12_**	60,0 ± 2,3	84,4 ± 3,1	98,0 ± 0,0	24,7 ± 2,3	54,0 ± 2,2	100,0 ± 0,0
**n-C_14_**	59,0 ± 2,5	82,3 ± 2,8	96,6 ± 0,0	27,3 ± 3,9	59,7 ± 2,3	100,0 ± 0,0
**n-C_16_**	60,7 ± 0,0	79,4 ± 0,0	93,6 ± 2,0	29,5 ± 9,8	65,2 ± 1,8	100,0 ± 0,0
**n-C_18_**	67,0 ± 2,0	86,4 ± 2,9	99,2 ± 0,2	34,0 ± 3,3	68,5 ± 0,9	100,0 ± 0,0
**n-C_20_**	67,9 ± 1,9	82,9 ± 1,8	93,7 ± 0,3	40,6 ± 2,0	72,9 ± 0,2	100,0 ± 0,0
**n-C_22_**	70,4 ± 1,8	85,6 ± 1,2	97,9 ± 2,1	38,8 ± 1,7	75,2 ± 0,9	100,0 ± 0,0
**n-C_24_**	71,8 ± 0,7	87,1 ± 2,2	100,0 ± 0,0	42,7 ± 2,9	77,5 ± 0,3	100,0 ± 0,0

After cryopreservation and storage at -20°C for 6 months, ENZ-G1 and ENZ-G2 displayed G1 and G2 hydrocarbon biodegradation rates, extents and specificity as well as cultivable bacterial biomass growth profiles comparable to those observed for the same consortia under the same culture conditions before storage (Figure 1). In particular, average degradation rates of 143.9 ± 6.4 and 162.7 ± 2.2 mg of overall hydrocarbons depleted l^-1 ^day^-1 ^were detected after four days of incubation in ENZ-G1 and ENZ-G2 cultures, respectively, and final depletions of overall applied hydrocarbons of 90.7 ± 0.6% (ENZ-G1) and 88.2 ± 0.4% (ENZ-G2) were observed at the end of incubation (Figure 1A). In addition, the concentration of the bacterial biomass able to grow on TSA medium increased from 7.6 × 10^6 ^± 1.8 × 10^6 ^to 5.2 × 10^8 ^± 4.4 × 10^7 ^CFU ml^-1 ^and from 1.2 × 10^7 ^± 3.2 × 10^5 ^to 5.7 × 10^8 ^± 3.6 × 10^7 ^CFU ml^-1 ^during the first 3 days of incubation in ENZ-G1 and ENZ-G2 cultures, respectively (Figure 1B). A careful observation of the morphology of colonies grown on TSA plates after 5 days of incubation indicated that both consortia were composed by seven bacterial components, two of which were numerically predominant on the others.

### Characterization of ENZ-G1 and ENZ-G2

The bacterial composition of ENZ-G1 and ENZ-G2 was characterized after the 5^th ^enrichment cycle by using two cultivation-independent methods based on 16S rRNA genes screening, i.e. 16S rDNA clonal libraries and PCR-DGGE fingerprinting of community 16S rDNA. Only three different OTUs were detected in each clonal library. Both clonal libraries from ENZ-G1 and ENZ-G2 cultures were dominated by OTU1, that represented 96% of screened clones and was 99% identical to 16S rRNA gene sequence of *Acinetobacter baumannii *strain NBRAJG89. OTU2 and OTU3 showed their highest sequence similarity to a *Paracoccus versutus *strain and represented 2% each of ENZ-G1 clones. On the contrary, 4% of ENZ-G2 clones were equally represented by OTU4, having 95% sequence identity with an uncultured Gammaproteobacterium, and by OTU5, having 99% sequence similarity with an uncultured *Achromobacter *sp. clone (Table 2).

**Table 2:** Phylotypes detected in ENZ-G1 and ENZ-G2 consortia by clone library screening and DGGE analysis of 16S rRNA genes

Consortium	Phylotype	Phylogenetic affiliation	Closest relative [GenBank Accession number]	Identity	Corresponding isolate
ENZ-G1 & ENZ-G2	DGGE band 1	Bacteroidetes	*Chryseobacterium taichungense *type strain CC-TWGS1-8 [AJ843132]	98%	ENZ2

ENZ-G2	DGGE band 4	α-proteobacteria	*Sphingopyxis taejonensis *Type strain JSS-54 [AF131297]	99%	-
ENZ-G1	Clone OTU2		*Paracoccus versutus *strain Tcn3 [AF437875]	99%	-
ENZ-G1	Clone OTU3		*Paracoccus versutus *strain Tcn3 [AF437875]	89%	-

ENZ-G1 & ENZ-G2	DGGE band 5	β-proteobacteria	*Alcaligenes *sp. NyZ215 [EF540877]	100%	ENZ3
ENZ-G2 & ENZ-G2	Clone OTU5		Uncultured *Achromobacter *sp. clone OUT11 [EU372808]	99%	-

ENZ-G1 & ENZ-G2	Clone OTU1	γ-proteobacteria	*Acinetobacter baumannii *strain NBRAJG89 [EU661706]	99%	ENZ1
ENZ-G1 & ENZ-G2	DGGE band 2		*Acinetobacter baumannii *strain NBRAJG89 [EU661706]	99%	ENZ1
ENZ-G1 & ENZ-G2	DGGE band 3		*Pseudomonas stutzeri *strain SA1 [DQ059546]	99%	ENZ4
ENZ-G1 & ENZ-G2	DGGE band 6		*Stenotrophomonas acidaminiphila *strain NK 2.Ha-5 [EU352763]	95%	ENZ6
ENZ-G2	Clone OTU4		Uncultured bacterium clone BANW729 [DQ264639]	95%	-

**Figure 3:**
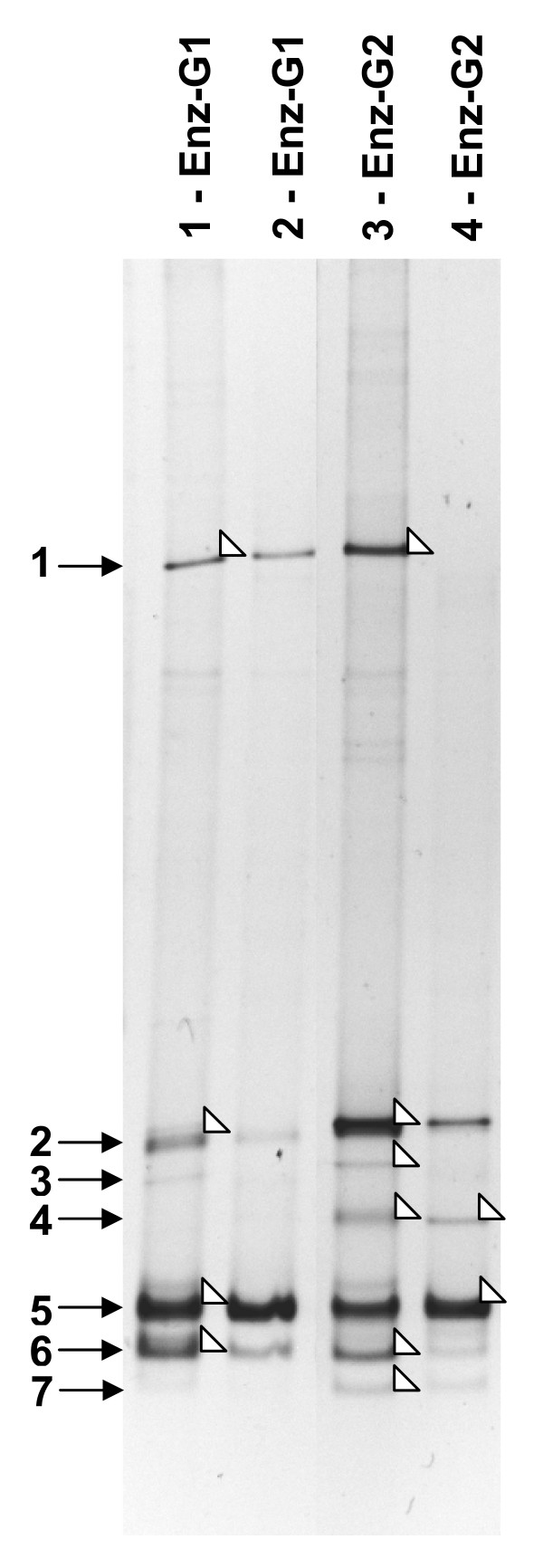
**DGGE analysis of ENZ-G1 and ENZ-G2 consortia**. 16S rDNA-DGGE profiles of diesel fuel degrading ENZ-G1 and ENZ-G2 consortia enriched from ENZYVEBA before (lanes 1 and 3) and after a six-month chryopreservation at -20°C (lanes 2 and 4). Numbered black arrows on left side indicate phylotypes detected as described in the text. White arrows indicate excised bands.

On the other hand, according to DGGE analysis 7 main phylotypes apparently occurred in ENZ-G1 and ENZ-G2 cultures (Figure 3, lanes 1 and 3). ENZ-G1 consisted of phylotypes 1, 2, 3, 5 and 6, whereas ENZ-G2 comprised all the 7 phylotypes. Band excision and sequencing revealed that phylotype 1 belonged to *Chryseobacterium *genus of Bacteroidetes, phylotypes 2, 3 and 6 to the genera *Acinetobacter*, *Pseudomonas *and *Stenotrophomonas *of Gammaproteobacteria, respectively, phylotype 4 to the *Sphingopyxis *genus of Alphaproteobacteria and phylotype 5 to the genus *Achromobacter *of Betaproteobacteria (Table 2). Repeated attempts to PCR-amplify and sequence the DGGE band corresponding to phylotype 7 were unsuccessful.

DGGE analysis repeated on ENZ-G1 and ENZ-G2 after cryotreatment and storage at -20°C evidenced the persistence of almost all the major components of the consortium (Figure 3). Only phylotypes 1 and 3 became undetectable in DGGE profiles of ENZ-G2 after storage and three sub-culturing steps (Figure 3, lane 4). On the other hand, phylotypes 2, 5 and 6 exhibited the highest relative band intensity in all profiles of both ENZ-G1 and ENZ-G2, thus suggesting they were the dominant members of both communities. Remarkably, the *Acinetobacter *genus represented by DGGE phylotype 2 was also the most abundant according to clone library screening (OTU1).

### Isolation of cultivable members of ENZ-G1 and ENZ-G2 consortia

**Figure 4:**
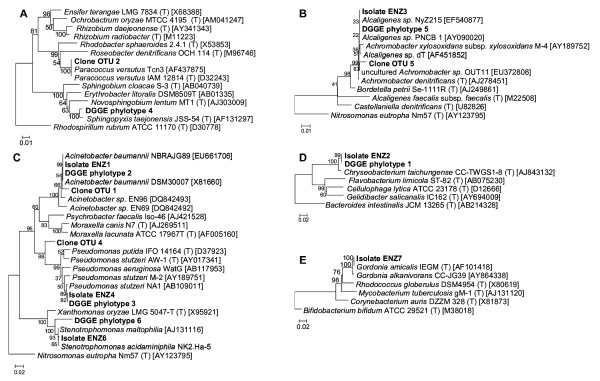
**Phylogenetic placement of bacterial isolates, DGGE bands and clones obtained from ENZ-G1 and ENZ-G2 consortia**. Neighbour joining phylogenetic trees based on 16S rDNA sequences. Bootstrap values were calculated on 1000 iterations. A: Alphaproteobacteria; B: Betaproteobacteria, C: Gammaproteobacteria; D: Bacteroidetes; E: Actinobacteria.

ENZ-G1 and ENZ-G2 plated on TSA displayed 6 and 7 different types of bacterial colonies, respectively. Over forty isolates were obtained from plates of ENZ-G1 or ENZ-G2. Those having the same colony morphology generated the same ARDRA profile. Thus 16S rRNA gene of one isolate for each unique restriction profile was sequenced for phylogenetic identification. Six pure cultures of bacteria, namely ENZ1 to ENZ6, were thus obtained from both ENZ-G1 and ENZ-G2 plated on TSA medium, and a seventh isolate (ENZ7) was obtained only from ENZ-G2; each isolate was transferred at least twice in liquid MMM with G1 or G2 at 1 g l^-1^. One isolate, i.e. ENZ5, obtained from either ENZ-G1 and ENZ-G2, failed to grow at the second transfer. All the others bacterial isolates grew very poorly and slowly. Isolates ENZ1, ENZ4 and ENZ6 were placed in the genera *Acinetobacter*, *Pseudomonas *and *Stenotrophomonas *of Gammaproteobacteria, isolate ENZ3 in the genus *Achromobacter *of Betaproteobacteria, isolate ENZ2 into the genus *Chryseobacterium *of Bacteroidetes and isolate ENZ7 in the genus *Gordonia *of Actinobacteria (Table 3). Remarkably, 16S RNA gene sequence of isolate ENZ1 (*Acinetobacter baumannii*) was highly similar to that of DGGE phylotype 2 and to OTU 1 identified in clonal libraries; whereas isolate ENZ2 (*Chryseobacterium taichungense*) was highly similar to DGGE phylotype 1, isolate ENZ3 (*Alcaligenes *sp.) to DGGE phylotype 5, isolate ENZ4 (*Pseudomonas stutzeri*) to DGGE band 3 and isolate ENZ6 (*Stenotrophomonas acidiminiphila*) to DGGE phylotype 6 (Figure 4). Conversely, 16S rRNA sequence of isolate ENZ7 did not correspond to any of the phylotypes detected by DGGE or in clonal libraries of ENZ-G1 and ENZ-G2.

**Table 3:** Phylogenetic identification of isolates obtained from ENZ-G1 and ENZ-G2 consortia

Consortium	Isolate	Closest Relative [GenBank Accession number]	Identity
ENZ-G1 & ENZ-G2	ENZ1	*Acinetobacter baumannii *DSM 30007 (T) [X81660]	100%
ENZ-G1 & ENZ-G2	ENZ2	*Chryseobacterium taichungense *strain CC-TWGS1-8 [AJ843132]	98%
ENZ-G1 & ENZ-G2	ENZ3	*Alcaligenes *sp. NyZ215 [EF540877]	100%
ENZ-G1 & ENZ-G2	ENZ4	*Pseudomonas stutzeri *strain SA1 [DQ059546]	99%
ENZ-G1 & ENZ-G2	ENZ6	*Stenotrophomonas acidiminiphila *strain D3 [EU301768]	99%
ENZ-G2	ENZ7	*Gordonia amicalis *Type strain IEGM [AF101418]	100%
ENZ-G1 & ENZ-G2	ENZ8	*Trametes gibbosa *strain 911030 [EU1620578]	100%

**Figure 5:**
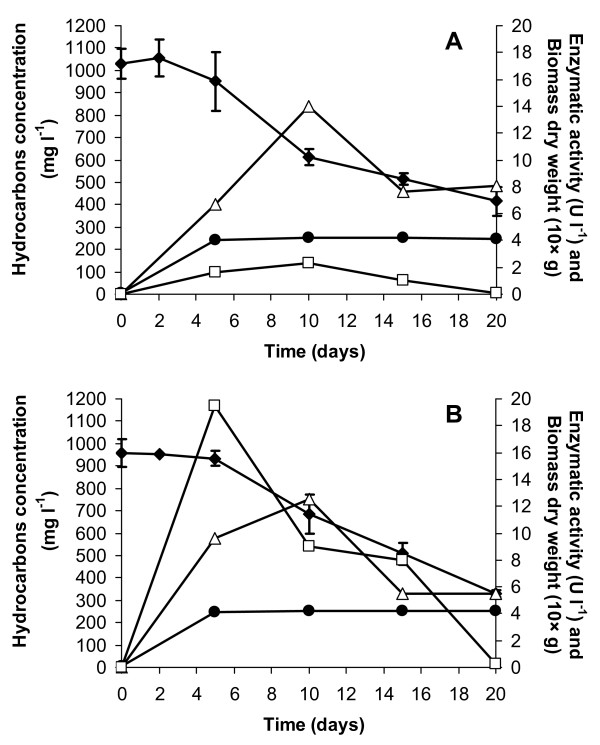
**Growth of the fungal isolate *Trametes gibbosa *on diesel fuels**. Growth of the fungal isolate *Trametes gibbosa *on MMM medium amended with G1 (A) and G2 (B) diesel fuels as the sole carbon and energy source. Black diamond: Hydrocarbons concentration; Black circle: dry weight biomass; White triangle: Laccase activity; White square: Mn-dependent peroxidase activity.

None of the characterized isolates degraded efficiently and in a reproducible manner G1 or G2 hydrocarbons under the liquid culture conditions described above adopted for determining ENZ-G1 and ENZ-G2 biodegradation potential (data not shown). No fungal colonies were observed to grow on TSA plates inoculated with ENZ-G1 and ENZ-G2 after 10 days of incubation. However, the use of MEA plates evidenced that both consortia contained fungi which displayed the same colony morphology (hyaline sterile mycelium). Some fungal isolates were obtained from ENZ-G1 or ENZ-G2 inoculated MEA plates but all of them were 100% identical to ITS gene sequences of several *Trametes gibbosa *strains. One of the fungal isolates, designated as ENZ8, was assayed for its ability to degrade G1 and G2 under the shaken flask batch conditions adopted for determining ENZ-G1 and ENZ-G2 biodegradation potential. It was found to remove 60% and 66% of overall applied G1 and G2 hydrocarbons, respectively, in 20 days of incubation (Figure 5). No GC-FID detectable intermediates were observed to accumulate in the cultures throughout their incubation. The fungal biomass grew rapidly during the first 5 days of incubation on G1 and G2 hydrocarbons, by increasing from 0.01 to about 0.40 g of dry biomass per 20 ml of culture medium (Figure 5). Extracellular ligninolytic enzyme activities were detected throughout the experiment, although generally at concentrations which were low and changed with the sampling time. A comparable laccase activity was detected in the culture medium throughout incubation on both G1 and G2, with a maximum activity of 14.0 and 12.5 Ul^-1^, respectively, at the 10^th ^day of incubation (Figure 5). Mn- peroxidase activity was also detected when ENZ8 was grown on G2 hydrocarbons, in particular after 5 days on incubation (19.2 Ul^-1^, Figure 5B), whereas the same enzymatic activity remained below 2.5 Ul^-1^during incubation with G1 (Figure 5A). Mn-independent peroxidase and lignino peroxidase activities were never detected.

## Discussion

Despite the majority of hydrocarbon constituents of diesel fuels are biodegraded by several microorganisms commonly occurring in soil each capable of breaking down a specific group of molecules [1], the bioremediation of soils and sites contaminated by diesel fuels is often limited by the poor biodiversity of indigenous microflora and/or the scarcity of indigenous specialized microbes with the complementary substrate specificity required to degrade the different hydrocarbons occurring at the contaminated site [7,29]. ENZYVEBA, a complex source of bacteria and fungi obtained through the prolonged stabilization of high quality organic wastes, was recently found capable of enhancing the aerobic bioremediation of a pristine soil freshly contaminated by diesel fuels, by providing both exogenous specialized microorganisms and nutrients [16]. In the present work, two microbial consortia, i.e., ENZ-G1 and ENZ-G2, able to grow on G1 or G2 in the absence of other carbon and energy sources, were obtained from ENZYVEBA through a conventional enrichment procedure and deeply characterized. In the view of employing them in bioaugmentation strategies of diesel fuels impacted sites, their ability to retain their biodegradation potential and community composition after cryopreservation was also investigated.

Both ENZ-G1 and ENZ-G2 exhibited high diesel fuel hydrocarbons biodegradation rates and yields, being able to remove about 90% of the applied 1 g l^-1 ^of G1 and G2, respectively, in 10 days of shaken batch aerobic culture incubation (Figure 1A). A comparable hydrocarbons biodegradation activity was displayed by both ENZ-G1 and ENZ-G2 after cryopreservation and storage at -20°C for six months (Figure 1A). The two consortia also displayed a similar biodegradation specificity towards G1 and G2 hydrocarbons, which were biodegraded simultaneously and with rates and extents which were only slightly dependent on the hydrocarbon molecular weight (Figure 2, Table 1). No GC-FID detectable intermediates were observed in the culture medium and this suggests that the depleted hydrocarbons underwent mineralization. These findings are in substantial agreement with those already reported in the literature for aerobic bacteria growing on complex mixtures of aliphatic hydrocarbons [30-32]. Slightly higher biodegradation rates were observed in ENZ-G2 cultures, probably for the G2 content of chemical additives with detergent properties able to increase, as already hypothesized by Di Toro et al. [16], the bioavailability and, in turn, the biodegradation of G2 hydrocarbons. However, such a finding might also be ascribed to the slightly higher biodiversity of ENZ-G2 consortium with respect to ENZ-G1 (Figure 3). Indeed, ENZ-G1 and ENZ-G2 display a similar but not identical community structure. Both cultures include members of Bacteriodetes (*Chryseobacterium taichungense*), Gammaproteobacteria (*Acinetobacter baumannii*, *Psudomonas stutzeri *and *Stenotrophomonas acidaminiphila*), Betaproteobacteria (*Alcaligenes *sp.), Actinomycetes (*Gordonia amicalis*) and Alphaproteobacteria; however, the latter group is represented by *Paracoccus versutus *in ENZ-G1, and by *Sphingopyxis taejonensis *in ENZ-G2 (Tables 2 and 3). With the exception of the Alphaproteobacteria strains, all members of the two microbial consortia identified with cultivation-independent techniques could be isolated from the same consortia and cultivated on mineral medium with G1 or G2. On the contrary, *Paracoccus *and *Sphingopyxis *strains were never isolated in pure culture under the same conditions probably because of their poor cultivability [33] or/and low occurrence in ENZ-G1 and ENZ-G2 consortia. Differently, the *Gordonia *strain isolated from both consortia was not detected via 16S rRNA gene clone library and DGGE analysis, probably because of the difficulties to brake up cells and obtain DNA from Actinomycetes with the DNA extraction protocol employed in this study relying on freeze and thaw cell lysis [34]. Several strains of ENZ-G1 and ENZ-G2 belong to bacterial species already reported in the literature for their ability to degrade aliphatic hydrocarbons. Indeed, several *Acinetobacter*, *Pseudomonas*, *Stenotrophomonas*, *Alcaligenes*, and *Gordonia *strains have been reported to grow on and/or degrade aliphatic and/or aromatic hydrocarbons or to enrich in diesel- and oily-sludge-contaminated soils [6,9,12,35-40]. However, ENZ-G1 and ENZ-G2 bacterial biodiversity is higher than that displayed by any other hydrocarbon-degrading mixed culture reported so far in literature. Further, to our knowledge, this is the first study in which *Paracoccus *and *Sphingopyxis *strains are reported as members of aliphatic hydrocarbon-biodegrading consortia. The composition of ENZ-G1 and ENZ-G2 did not change significantly after cryopreservation. However, importantly, none of the bacterial isolates were able to stably and remarkably grow on G1 or G2 hydrocarbons in liquid cultures of MMM supplemented with G1 or G2 at 1 g l^-1^. This was quite surprising and can be ascribed to the inability of isolates to singly face the toxicity of the complex hydrocarbons mixture occurring in diesel fuel [41].

Such an unexpected finding induced new investigations addressed to search for the occurrence of fungi in the two consortia and fungal strains belonging to the *Trametes gibbosa *species able to grow on G1 and G2 hydrocarbons in shaken cultures of MMM were obtained from both ENZ-G1 and ENZ-G2. This was a quite surprising evidence as the enrichment procedure adopted for obtaining the two consortia was specific for bacteria, in terms of medium composition, mixing and incubation conditions adopted, and one of the most commonly applied for the selection of prominent hydrocarbon degrading mixed cultures described in the literature during the last two decades [22,42-45]. Several fungi are known to have the capability of degrading persistent pollutants. The microbial degradation of hydrocarbons by ligninolytic fungi has been intensively studied during the past few years [46] because they produce extracellular enzymes with very low substrate specificity, making them suitable for degradation of different compounds. The genus *Trametes *is probably the most actively investigated in the phylum of Basidiomycota for ligninolytic enzyme formation and use in a wide range of analytical, industrial, and environmental applications [47]. *Trametes *spp. are well known degraders of different organopollutants and *T. gibbosa *in particular has already been reported for its ability to degrade different dyes [48].

The co-presence of bacteria and fungi appear essential for the functionality of the consortium, as demonstrated by our data and confirm what already stated by other authors that showed that the mineralization of hydrocarbons in nature may require the combined efforts of both fungi and bacteria [49].

Thus, ENZ-G1 and ENZ-G2 display an overall biodiversity higher than those of other diesel fuel mineralizing aerobic consortia already reported in the literature and are the first ones consisting of stable consortium of bacteria and the fungus *Trametes gibbosa*, and probably for this ENZ-G1 and ENZ-G2 are the consortia with the broadest specificity and highest biodegradation potential among the diesel fuel biodegrading cultures reported so far in the literature.

## Conclusions

ENZ-G1 and ENZ-G2 are two new aerobic microbial consortia capable of rapidly and extensively degrading linear paraffins from n-C10 to n-C24 of two different commercial largely used diesel fuels under well defined laboratory conditions and this thanks to their richness of quite diverse microbes, such as bacteria and the fungus *Trametes gibbosa*. Their remarkable and reproducible biodegradation potential together with their resistance to chryopreservation make ENZ-G1 and ENZ-G2 consortia very interesting candidates for bioaugmentation operations on diesel fuels impacted sites. Given their very similar composition and biodegradation activity both these mixed cultures are equally promising in the bioaugmentation practice of soils contaminated by diesel fuels. Laboratory scale studies addressed to test both cultures on actual site soils are currently in progress. However, the main lesson learnt in this study is that fungi can be enriched together with bacteria through the enrichment procedures conventionally adopted for obtaining consortia of highly specialized aerobic bacteria. Thus, the occurrence of fungi in mixed bacterial cultures previously obtained with the same procedure cannot be excluded.

## Methods

### Source of microorganisms and chemicals

ENZYVEBA, which is a partially characterized stable consortium of aerobic and anaerobic bacteria and fungi [25-28], was employed as the source of diesel fuel hydrocarbon-degrading microorganisms. It was obtained by MARCOPOLO ENGINEERING S.p.A. (Italy) from selected organic matter through a twenty-years culture enrichment process (MPE Mesen Patented - No. 01286120) and was partially characterized through conventional and molecular procedures [25,28]. A 30% w/v suspension of ENZYVEBA in sterile water was incubated at 37°C and 100 rpm for 24 hrs and used to inoculate the first enrichment culture.

Solvents and chemical reagents were obtained from Sigma-Aldrich and Carlo Erba (Milan, Italy) whereas the components of media used for growing microorganisms, i.e., mineral minimal medium (MMM), Tryptic Soy Agar (TSA); and Malt Extract Agar (MEA) and glycerol, were purchased from Biolife Italia (Milan, Italy). The piperacillin/tazobactam combination and gentamicin sulfate were purchased at pharmacies. Molecular biology reagents were purchased from Invitrogen (Milan, Italy).

### Diesel fuels employed

Two different types of commercially available diesel fuels taken from a Petroleum gas station, i.e. Diesel and HiQ Diesel, herewith designated as G1 and G2 respectively, were employed as the sole source of carbon and energy for enriching two distinct diesel fuel-degrading microbial populations from ENZYVEBA.

G1 and G2 have very similar distillation temperatures, viscosity and density but differ significantly in the content of sulfur and additives with detergent, antifoaming and anticorrosion properties as well as in cetane number. In particular, G2 has, with respect to G1, a lower sulfur content (10 mg kg^-1 ^versus 50 mg kg^-1^), a higher cetane number (53 versus 51), and a content of additives with lubricant properties (data not shown).

### Enrichment and isolation of diesel fuel-degrading cultures from ENZYVEBA

Two parallel consortia of aerobic microbes able to grow on G1 or G2 diesel hydrocarbons were enriched from ENZYVEBA by using 1.0 l baffled Erlenmeyer flasks equipped with Teflon-coated screw caps containing 0.3 l of minimal mineral medium (MMM) amended with 20 mg l^-1 ^yeast extract (as a source of vitamins) and 1 g l^-1 ^G1 (or G2) as the main energy and carbon source. In a first phase, 2 identical flasks, one with G1 and the other with G2, were inoculated at 2% (v/v) with the water suspension of ENZYVEBA prepared as mentioned above and incubated at 30°C and 150 rpm for 10 days. The flasks were opened daily for 30 minutes to provide O_2 _by reducing hydrocarbons losses due to volatilization. At the end of the 10-days incubation, each culture was transferred at 2% v/v in a sterile flask containing, as before, 0.3 l of MMM with 20 mg l^-1 ^yeast extract and 1 g l^-1 ^G1 (or G2) and incubated for 10 days under the same conditions. Three other identical sequential transfers were performed of each of the two cultures, thus obtaining, after 5 culture enrichment cycles, two microbial consortia capable of growing on G1 and G2 diesel fuels, designated as ENZ-G1 and ENZ-G2, respectively. To characterize the main microbial features of the two consortia and assay their biodegradation potential, ENZ-G1 and ENZ-G2 were inoculated in duplicate into sterile MMM with their own diesel fuel and managed as follows. Seventeen 0.1 l baffled Erlenmeyer flasks, containing 20.0 ml of MMM medium amended with 20 mg l^-1 ^yeast extract and 1 g l^-1 ^G1 or G2 were inoculated with ENZ-G1 or ENZ-G2, respectively, and incubated at 30°C and 150 rpm for 10 days. On days 0, 1, 2, 3, 4, 6, 8 and 10, two flasks per each enriched culture were subjected to sampling (1.0 ml aliquot) for the analysis of total viable biomass concentration, and the remaining culture broth was extracted for qualitative and quantitative analysis of diesel fuel hydrocarbons. On day 10, an additional flask of each grown mixed culture was used for community DNA extraction and to prepare culture stocks in glycerol (20% v/v in MMM medium) that were frozen in liquid nitrogen and chryopreserved at -20°C. Sixteen controls were also set up according to the same procedure for ENZ-G1 and ENZ-G2, sterilized after inoculation and used to measure possible abiotic losses of hydrocarbons all along the experiment. After a six-month period of storage at -20°C, ENZ-G1 and ENZ-G2 were re-inoculated in MMM amended with 20 mg l^-1 ^yeast extract and 1 g l^-1 ^of their own diesel fuel and further sub-cultured for additional 3 cycles, as described above, in order to remove glycerol traces from the culture, and analyzed for hydrocarbon biodegradation and microbial community composition.

Bacterial isolates capable of growing on G1 and G2 were obtained from ENZ-G1 and ENZ-G2, respectively, by growing the two cultures on TSA agar plates, tooth-picking single colonies having different morphology and transferring them into liquid MMM supplemented with 20 mg l^-1 ^yeast extract and 1.0 g l^-1 ^G1 or G2 as the sole carbon and energy source. Each isolate was sub-cultured at least two times in the same medium under the same conditions. Purity of isolates was verified by streaking their liquid cultures on TSA plates. Isolates were grouped into ARDRA (Amplified Ribosomal DNA Restriction Analysis) Operational Taxonomic Units (OTUs) and phylogenetically identified as described below.

Fungi were also obtained from MEA agar plates supplemented with antibiotics (gentamicine 40 mg l^-1 ^and piperacilline plus tazobactam 100 mg l^-1^) and inoculated with ENZ-G1 and ENZ-G2, respectively. The fungal isolates were characterized via genetic analysis as reported below.

The ability of the bacterial isolates to grow on and biodegrade the diesel fuel used for their selection was studied by inoculating a cell suspension of each of them prepared in MMM in duplicate MMM cultures with 20 mg l^-1 ^yeast extract and 1 g l^-1 ^G1 (or G2) incubated at 30°C and 150 rpm for 10 days according to the approach adopted for quantifying the activity of ENZ-G1 and ENZ-G2 described above. To determine the diesel fuel biodegradation capability of fungal isolates, the same approach was used with the following modifications. Twenty-two 0.1 l baffled Erlenmeyer flasks, containing 20.0 ml of MMM medium amended with 20 mg l^-1 ^yeast extract and 1 g l^-1 ^G1 or G2, were inoculated with a cell suspension of the fungal isolate prepared in MMM and incubated at 30°C and 150 rpm for 20 days. On days 0, 2, 5, 10, 15 and 20, two flasks were used to extract and analyse qualitatively and quantitatively diesel fuel hydrocarbons. On days 0, 5, 10, 15 and 20, two additional flasks were subjected to sampling of a 0.5 ml aliquot for the analysis of ligninolitic enzyme activities and the remaining culture broth was used for fungal biomass dry weight determination. Killed controls were also set up and sacrificed on days 0 and 20 to measure possible abiotic losses of hydrocarbons.

### Analytical procedures

G1 and G2 hydrocarbons were batch extracted from MMM liquid cultures of both enriched and pure cultures with 1 volume of a mixture of hexane:acetone (9:1) and sonication for 10 min. The qualitative and quantitative analysis of G1 and G2 hydrocarbons occurring in the organic extracts were performed with a gas chromatograph (5890 series II), equipped with a HP-5 capillary column (30 m by 0.25 mm) and a flame ionization detector (FID) (Hewlett-Packard Co., Palo Alto, CA, USA). All runs were conducted under the following conditions: initial temperature, 60°C; isothermal for 1 min; temperature rate, 10°C min^-1^; final temperature, 320°C; isothermal for 5 min. The injector (splitless mode) was at 270°C, FID at 320°C; carrier gas (N_2_) flow rate was 60 ml min^-1 ^and the injected sample volume of 1 μl. GC peaks were characterized by injecting standards of target *n*-paraffins under the same conditions. Quantitative analysis of G1 and G2 linear hydrocarbons as well as of the overall G1 and G2 hydrocarbons was performed by running five-points linear calibrations of target *n*-paraffins (UltraScientific, N. Kingstown, RI, USA) and of both diesel fuels in the concentration range of 50-1000 mg l^-1 ^under the same analytical conditions. The biodegradation of G1 and G2 hydrocarbons was determined by performing duplicate analyses of the organic extracts obtained from each culture sacrificed at each sampling time and subtracting the abiotic losses quantified in the parallel sterile controls.

The concentration of the total aerobic cultivable bacteria was determined through the colony counting technique on TSA plates. It was expressed as the mean of colony forming units per ml counted on duplicate samples collected from the two duplicate flasks at each sampling time.

The fungal growth was evaluated as dried weight at each sampling time when the biomasses were filtered on filter paper (Whatman type 1), placed in an oven and dried at a temperature of 65°C for 24 h; then they were weighed and the dry weight of the fungal biomass was calculated.

Laccase activity was assayed at 25°C using 2.2'-azinobis (3-ethylbenzothiazoline-6-sulfonic acid) (ABTS) as substrate [50]. Mn- peroxidase and Mn-independent peroxidase activities were measured at 25°C using DMAB/MBTH [3-dimethylaminobenzoic acid/3-methyl-2-benzothiazolinone hydrazone hydrochloride] as substrates [51]. Lignino-peroxidase activity was assayed at 35°C using veratryl alcohol as substrate [52]. All the enzyme activities were expressed in International Units (IU), where 1 unit is defined as the amount of enzyme that oxidize 1 μmole of substrate in 1 minute.

### Genomic DNA extraction from mixed and pure cultures and bacterial and fungal gene amplification

Genomic DNA was extracted from the pellets resulting from centrifugation of 2.0 ml active mixed and pure bacterial cultures using a procedure modified from Kerkhof and Ward [53]. Cell pellets were suspended in 0.3 ml of a buffer solution containing 25 mM TRIS pH 8.0, 10 mM EDTA and 50 mM glucose. DNA extraction consisted of 8 freeze and thaw cycles in liquid nitrogen/water bath (37°C) followed by addition of 0.1 ml of the same buffer solution containing 5 mg ml^-1 ^of lysozyme and of 50 μl of 0.5 M EDTA, and by incubation on a rotary shaker at 37°C for 30 min. Fifty μl of 10% (w/v) SDS were then added and samples were immediately extracted twice with TRIS (pH 8.0) saturated phenol:chlorophorm:isoamylalcol (25:24:1). The mixture was vortexed vigorously to form an emulsion and centrifuged at 12500 × g for 3 min before removing the non-aqueous phase. DNA was then precipitated with 0.1 volumes sodium acetate 3 M pH 5.2 and 2 volumes 100% cold ethanol, pelleted at 12500 × g for 20 min and washed twice with 0.25 ml 70% cold ethanol at 12500 × g for 10 min. DNA pellets were air dried and resuspended in 50 μl deionized water. 16S rRNA gene PCR amplification was performed with a T-gradient Thermocycler (Biometra, Göttingen, Germany). Amplification reaction mixtures contained 1 × PCR buffer, 1.5 mM MgCl_2_, a 0.2 mM concentration of each dNTP, a 0.4 mM concentration of each primer, 2.0 U of *Taq *polymerase, and 2.0 μl of template DNA in a final volume of 50 μl. The primers pairs used were 27f (5'-AGA GTT TGA TCC TGG CTC AG-3') - 1495r (5'-GTT TAC CTT GTT ACG ACT T-3') [54] and GC-357f, containing a 40 bp GC-clamp, (5'-**CGC CCG CCG CGC CCC GCG CCC GGC CCG CCG CCC CCG CCC C**CC TAC GGG AGG CAG CAG-3') - 907r (5'-CCG TCA ATT CCT TTG AGT TT-3') [55], depending on the following processing of the PCR-amplified product. Reaction mixtures were held at 94°C for 2 min, followed by 30 cycles of amplification at 94°C for 30 s, 55°C for 30 s, and 72°C for 45 s and a final extension of 72°C for 10 min. Amplification products were analyzed in 1.0% (w/v) agarose gel stained with ethidium bromide.

Genomic DNA of fungal isolates was extracted using the DNeasy plant mini kit (Qiagen, Germany) according to the manufacturer's instructions. The ITS region of the nuclear ribosomal DNA (5.8S) was amplified using the forward primer ITS1F (5' CTTGGTCATTTAGAGGAAGTAA 3') [56] and the reverse primer ITS4 (5' TCCTCCGCTTATTGATATGC 3') [57]. The PCR reactions were performed in 50 μl volume. The DNA concentration was 30 ng ml^-1^. PCR took place under the following conditions: 95°C for 5 min; 94°C for 45 sec, 56°C for 45 sec, 72°C for 45 sec (35 cycles); 72°C for 7 min. The reaction was then maintained at 4°C. The resulting amplification products were analyzed by 1% (w/v) agarose gel electrophoresis.

### 16S rRNA genes clonal libraries

PCR-amplified 16S rRNA genes obtained from genomic DNA of mixed cultures with the primer pair 27F-1495r were cloned in pCR^®^4-TOPO^® ^cloning vector (Invitrogen, Paisley, UK) and the resulting plasmids were inserted in chemically competent One Shot^® ^TOP10 *E. coli *cells (Invitrogen, Paisley, UK) according to the manufacturer's instructions. Transformed cells were plated on LB agar plates containing 100 μg ml^-1 ^ampicillin and incubated overnight at 37°C. Fifty colonies from each clonal library were tooth-picked and grown overnight in liquid LB medium with 100 μg ml^-1 ^ampicillin. Library screening used plasmid extraction, PCR amplification of insert and digestion with the restriction enzyme AluI to identify unique banding patterns. Plasmids extractions used a modified alkaline lysis miniprep protocol [58]. PCR amplification of inserts was performed with primer pair 27f-1495r as described above in a final volume of 25 μl. Restriction reaction mixtures contained 1 × REact^® ^1 buffer (Invitrogen, Paisley, UK), approximately 0.5 μg of DNA and 2 U of AluI in a final volume of 20 μl. Restriction digestion was performed at 37°C for 6 hrs followed by enzyme inactivation at 65°C for 10 min. The restriction digests were separated in 2% (w/v) agarose gels in 0.5 × TBE buffer. Amplified inserts bearing a unique restriction banding pattern were sequenced with primer 27f as described below.

### DGGE analysis

PCR-amplified 16S rRNA genes obtained from genomic DNA of mixed cultures with the primer pair GC357f - 907r were separated on the basis of their melting behaviour by denaturing gradient gel electrophoresis (DGGE). DGGE fingerprint was performed with a D-Code apparatus (Bio-Rad, Milan, Italy) in a 7% (w/v) polyacrylamide gel (acrylamide-N, N'-methylenebisacrylamide, 37:1) in 1 × TAE with a denaturing gradient of 40% (top) to 55% (bottom) denaturant, where 100% denaturant is 7 M urea and 40% (v/v) formamide. The electrophoresis was run at 55 V for 16 hrs at 60°C. The gel was stained in a solution of 1 × SYBR-Green in 1 × TAE for 30 min and its image captured in UV transillumination with a digital camera supported by a Gel Doc apparatus (Bio-Rad, Milan, Italy). Bands were cut from the gel with a sterile scalpel and DNA was eluted by incubating the gel fragments for 16 hrs in 50 μl of sterile deionized water at 4°C. Two μl of the solution were then used as template to re-amplify the band fragment using the same primers without the GC-clamp (357f-907r) and the same PCR conditions described above. The obtained amplicons were then sequenced with primer 357f as described below.

### ARDRA

16S rRNA genes of pure cultures amplified with the primer pair 27f - 1495r were digested with the restriction enzyme AluI to identify unique restriction patterns as described for the clonal library screening. The restriction digests were separated in 2% (w/v) agarose gels in 0.5 × TBE buffer and isolates were grouped into Operational Taxonomic Units (OTUs) having unique restriction patterns. The PCR-amplified 16S rRNA gene of one representative of each OTU was sequenced with primer 27f as described below.

### Sequencing and phylogenetic analysis

Sequencing of bacterial 16S rRNA amplicons was performed after purification with EXOSAP (USB Corporation, Cleveland, Ohio, US) according to the manufacturer's instructions. Sequencing reactions and runs were performed by BMR Genomics (Padova, Italy).

For each 16S rDNA sequence, the most closely related sequence and the sequence of the most closely related cultured bacterial strain were retrieved from the GenBank database by using MEGABLAST and from the Ribosomal Database Project-II by using the SEQUENCE MATCH tool. The sequences were aligned with the CLUSTAL W program and phylogenetic trees were calculated with the neighbour-joining method. Bootstrap values were determined from 1000 iterations. The fungal ITS region was sequenced in both strands using the primers ITS1F and ITS4. Sequences were obtained with a rapid (24 h) automated capillary electrophoresis system CEQ 2000 (Beckman Coulter, USA). The ITS sequences were compared to the GenBank (NCBI) database by BlastN algorithm BLASTN 2.2.18+ (September-26-2008) to identify the isolate or reveal the closest known analogue.

## Competing interests

SDT, DT and AB received their salary from MARCOPOLO ENGINEERING Spa, which also provided ENZYVEBA and partially financed the research. The other authors declare that they have no competing interests.

## Authors' contributions

GZ participated in the design of the study, performed the molecular analysis of microbial communities and drafted the manuscript. SDT participated in the design of the study and performed the enrichment and isolation of bacterial cultures. DT performed the hydrocarbons biodegradation experiments. GCV carried out the isolation and characterization of the fungal isolate and participated in the manuscript preparation. AB commented on the manuscript. FF coordinated the research as well as the manuscript preparation. All the authors read and approved the final manuscript.
